# Honey bees consider larval nutritional status rather than genetic relatedness when selecting larvae for emergency queen rearing

**DOI:** 10.1038/s41598-018-25976-7

**Published:** 2018-05-16

**Authors:** Ramesh R. Sagili, Bradley N. Metz, Hannah M. Lucas, Priyadarshini Chakrabarti, Carolyn R. Breece

**Affiliations:** 1 Department of Horticulture, Oregon State University, Corvallis, OR 97331 USA; 2 Department of Entomology and Plant Pathology, North Carolina State University, Raleigh, NC 27695 USA

## Abstract

In honey bees and many other social insects, production of queens is a vital task, as colony fitness is dependent on queens. The factors considered by honey bee workers in selecting larvae to rear new queens during emergency queen rearing are poorly understood. Identifying these parameters is critical, both in an evolutionary and apicultural context. As female caste development in honey bees is dependent on larval diet (i.e. nutrition), we hypothesized that larval nutritional state is meticulously assessed and used by workers in selection of larvae for queen rearing. To test this hypothesis, we conducted a series of experiments manipulating the nutritional status of one day old larvae by depriving them of brood food for a four-hour period, and then allowing workers to choose larvae for rearing queens from nutritionally deprived and non-deprived larvae. We simultaneously investigated the role of genetic relatedness in selection of larvae for queen rearing. In all the experiments, significantly greater numbers of non-deprived larvae than deprived larvae were selected for queen rearing irrespective of genetic relatedness. Our results demonstrate that honey bees perceive the nutritional state of larvae and use that information when selecting larvae for rearing queens in the natural emergency queen replacement process.

## Introduction

The social structure of the honey bee colony is such that, ordinarily, reproduction is consolidated to the efforts of a single individual: the queen. She is the only member capable of laying fertilized eggs that develop into females (workers and new queens). Additionally, she is the longest-lived member of the colony, with a lifespan (1 to 8 years) that typically far exceeds that of the other honey bee castes^1,2^. As a young virgin, during the only mating event of her life, the queen will mate with 5 to 21 males over a few days^3–9^. This polyandrous mating leads to a distribution of multiple patrilines among the fertilized eggs (and therefore the adult females) that a queen produces^5,8^. Fertilized eggs develop into larvae that are totipotent for the first through third instars; they could become either queens or workers^10–12^. Determination of their development into one or the other is driven solely by diet^13–15^. Larvae that are fed large quantities of nutrient-rich royal jelly from a totipotent instar to pupation become queens; those that are fed comparatively nutrient-dilute brood food become workers^13–18^. This developmental fork in the road makes it possible for nurse bees to raise selected totipotent larvae as queens if colony conditions are appropriate ^17,19–23^.

Honey bee colonies rear new queens (i) to replace dead/missing queens or queens that are no longer laying eggs (emergency queens); (ii) to replace injured, old or diseased queens that are not productive (supersedure queens); and (iii) in preparation for reproduction at the colony level even when the current queen is laying well (swarm queens)^24–27^. A colony that is unsuccessful in their attempts to rear a queen, in any queen rearing context, will eventually die without human intervention.

If the current queen has died or stopped laying eggs, emergency queens must be reared^28^. Thus, workers select eggs or totipotent larvae, in worker cells, to raise as queens. Selection is commonly signified by the modification of that worker cell into a vertically oriented, peanut-shaped queen cell^17,29,30^. Once selected, the larva is provisioned with royal jelly. Emergency queen rearing is, as the name suggests, of great urgency. The colony only has about 6 days after the last egg was laid to begin rearing new queens. After that time, the last eggs laid by the queen will have grown into larvae too old to reliably develop into fully functional queens (3 days as eggs and 3 days as totipotent larvae)^10,17,31^. The size, shape and orientation of a queen cell is a strong cue triggering queen rearing behavior (i.e. providing large quantities of royal jelly to the larvae inside)^17,28^.

Previous studies have shown that honey bee workers can recognize the brood and differentiate the castes and developmental stages of larvae^32–35^. Past work suggests that a contact surface pheromone is the major larval recognition signal in honey bees, and worker bees can differentiate hungry larvae from well-fed larvae^34,35^, potentially via detection of the volatile pheromone E-β-ocimene produced by hungry larvae^36^. But there is a significant gap in knowledge about the criteria that worker bees use in selecting larvae for rearing emergency queens. Much of the literature on this subject has focused on tests of nepotism and kin discrimination^37–44^. However, the experimental design of most studies supporting such a theory involved highly artificial conditions and methodological concerns^42,45–47^. Nepotism and kin discrimination are generally considered improbable in a colony comprised of a naturally occurring distribution of multiple patrilines^42,44,46^ and experimental support for kin selection in queen rearing is weak at best^40,43,47–51^. Only recently has there been exploration into other potential cues in larvae selection for queen rearing. These recent studies have demonstrated that resource availability affected kin selection of larvae^52^, larvae originating from eggs with greater weights were preferentially selected for queen rearing^53^ and larval cell location in the brood nest influenced emergency queen cell construction^30^. Nonetheless, much of it is yet to be understood. We hypothesized that because larval diet is the all-important determinant of whether a female larva develops into a queen, the nutritional state of larvae may, likewise, affect their selection for queen rearing. Here we tested the premise that well-fed larvae will be preferentially selected over their poorly nourished cohorts for queen rearing. To test this hypothesis we conducted a series of experiments manipulating the nutritional status of one-day-old larvae by depriving them of brood food for a period of 4 hours.

Previous studies on nepotism and kin discrimination primarily used a method called grafting to measure queen rearing^50,54^. Grafting larvae into large, vertically oriented artificial queen cells is a well-established method of mass queen rearing used in apiculture since the late 1800’s^55–57^. At least one previous study^50^ recognized a significant difference in the choices bees make when selecting larvae in natural queen rearing versus maintaining queen rearing in artificially grafted larvae. In natural emergency queen rearing, nurse bees select individual larvae or eggs present in the worker cells and modify the existing wax cell into a queen cell. The vertical orientation of a queen cell is a strong cue triggering queen rearing behaviors^17^. It is therefore reasonable to hypothesize that, in any examination of emergency queen larval selection, natural queen rearing and artificially grafted queen rearing methods may yield significantly different results. We tested this hypothesis by manipulating the nutritional status of totipotent larvae and rearing them in both a natural queen rearing environment and an artificial queen rearing environment (grafting).

In studies examining the cues honey bees use to make decisions about the acceptance of new individuals into the hive, nest-mate recognition, when to initiate foraging, the presence of the queen, queen rearing and a myriad other aspects of colony function, chemical mediators have commonly been observed^58,59^. When introducing foreign individuals to a colony, combined odors of their natal colony are important^60^. These odors come from endogenously produced chemicals and from food sources and other material adsorbed into the surrounding wax comb^61^. Because our experimental methods included introducing larvae in brood comb into unrelated colonies, it was necessary to observe the potential effects of comb odor on larval choice for queen rearing as well.

To address all the questions/hypotheses discussed above, we conducted the following series of experiments. The first experiment (variation in nursing times between same age larvae) was conducted to examine if there were some larvae that were not fed for at least 4 hours in a colony and also to observe variation in feeding times between larvae. The second experiment was performed to investigate the behavior of nurse bees towards deprived (not fed) versus non-deprived (fed) larvae. In the third experiment, we attempted to resolve a critical aspect of the methodology used to test the effects of larval nutritional deprivation on selection of larvae for queen rearing by measuring selection under natural colony conditions versus artificial grafting. Further, in the fourth experiment (comb odor experiment), we tested whether a 12-hour placement of frame (comb) in an unrelated colony would mask the comb odor of the parental colony. This experiment was necessary to discount any comb odor bias during larval selection for queen rearing. Finally, in the fifth experiment, we tested the effects of both relatedness and nutritional status of larvae in selection of larvae for queen rearing. We used colonies headed by open mated queens and colonies headed by super sister, single-drone inseminated queens for this experiment.

## Results

### Experiment 1. Variation in feeding frequency and duration between same age larvae

We used video recordings of observation hives to quantify various aspects of nurse bee visits at 2-day old (young) and 5-day old (older) larvae. For the young larvae (2-day old) we observed visitations of nurse bees to 75 larvae in each of the 4 observation hives (four-frame observation hives) to see if any larvae were not fed (neglected) at all during the 4 hour observation period. In another separate experiment we used 5 two-frame observation hives to measure the total amount of time spent by nurse bees feeding selected individual larvae that were older (5-day old) during a one hour observation period. The overall goal of experiment 1 was to characterize how long a given larva might be neglected and, conversely, for how much time a larva may be fed.

#### Young larvae

Upon analysis of the nurse bee visitation data for each larvae, it was found that 6 larvae out of a total of 75 (8%), 4 larvae out of a total of 75 (5%), 6 larvae out of a total of 75 (8%) and 3 larvae out of a total of 75 (4%) were not visited by nurse bees at all during the observation period (4 hours) in the observation hives 1, 2, 3 and 4 respectively.

#### Older larvae

Distribution of larval feeding times was found to be significantly different from uniform distribution, with a clear right skew (Z = −4.73, df = 124, p < 0.0001). The mean total feeding time per larva was 6.82 ± 0.68 minutes. The maximum interval between feeding bouts was 36.57 minutes; the median interval between feeding bouts was 3.86 minutes. Similar to the previous experiment (1a), we found that approximately 10% of the total observed larvae were not visited at all during the observation period.

### Experiment 2. Feeding response to nutritionally deprived and non-deprived larvae

To examine how temporary nutritional deprivation of larvae may influence the ways in which nurse bees allocate their efforts and resources among totipotent larvae, we established observation hives in which we artificially deprived approx. 500 larvae of brood food while allowing approx. 500 larvae to be fed, and then measured nurse bee response to those larvae. We measured time to first feeding, time to first inspection, total number of feeding bouts, total number of inspections, duration of each feeding bout and total time fed for deprived and non-deprived larvae. When there was no significant difference between replicates, we pooled the data from all replicates within a treatment group for analysis.

**Figure 1:**
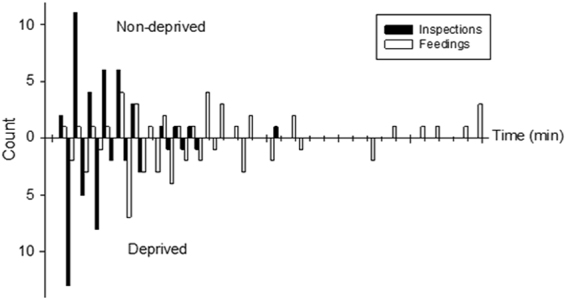
Paired histogram of the distribution in time to first inspection (black bars) and the time to first feeding (white bars) with the non-deprived larvae occupying the positive Y-axis and the deprived larvae the negative Y-axis are shown in this figure. Both time to first inspection and time to first feeding were significantly (p < 0.05) shorter for the deprived larvae compared to the non-deprived larvae. The distribution time and time to first feeding were observed in 6 larvae from each deprived and non-deprived group for a total 30 minutes per larva (N = 2).

**Figure 2:**
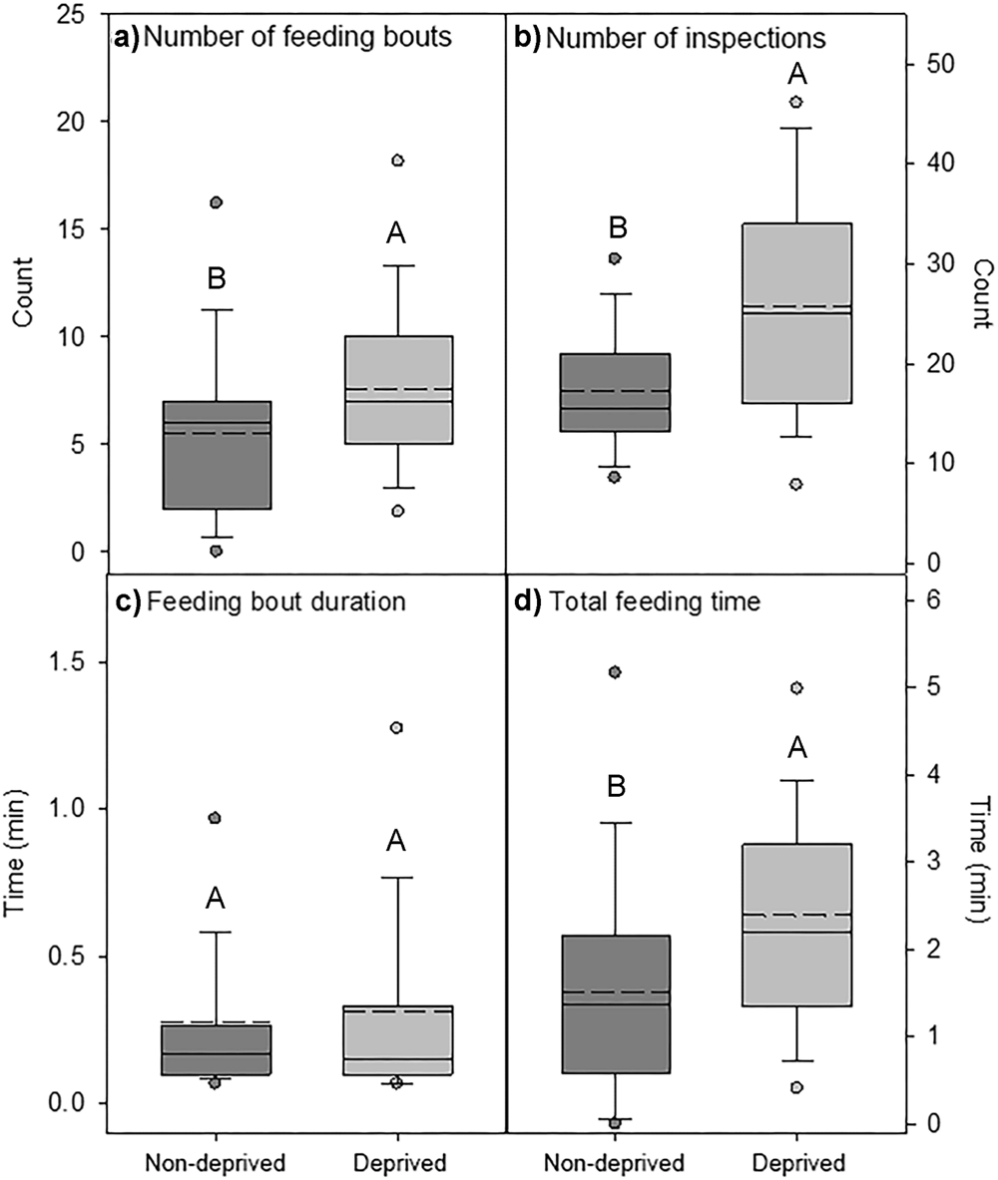
Figure shows nursing responses to side-by-side patches of deprived (light grey) and non-deprived (dark grey) larvae. Nurses generally attended deprived larvae with greater frequency over the observation period with a significantly greater number of (**a**) feedings (p = 0.001), (**b**) inspections (p < 0.001), and (**d**) longer total feeding time (p = 0.011). (**c**) Duration of individual feeding bouts were not significantly different between treatments (p = 0.881). Nursing responses were analyzed for a total of 6 larvae from each of the deprived and non-deprived groups and the data was pooled for each group (N = 2). For all variables with the same letter (A or B), the difference between the means is not statistically significant.

The time to first feeding was significantly less for the deprived larvae than for the non-deprived larvae (F_1,5_ = 6.853, p = 0.047). Also, time to first inspection was significantly shorter for deprived larvae compared to non-deprived larvae (F_1,70_ = 4.988, p = 0.029). The distribution of time to first inspection and to first feeding in the two groups have been described in Fig. 1. Further, the deprived larvae were fed (χ^2^ = 11.602, df = 1, p = 0.001; Fig. 2a) and inspected (χ^2^ = 60.055, df = 1, p < 0.001; Fig. 2b) a significantly greater number of times throughout the observation period compared to the non-deprived larvae. The mean duration of feeding bouts was not significantly different between the two treatments (Z_4,70_ = −0.150; p = 0.881, Fig. 2c). Finally, it was also observed that the deprived larvae were fed for a significantly greater total amount of time than the non-deprived larvae (F_1,70_ = 6.892, p = 0.011; Fig. 2d).

### Experiment 3. Effect of queen rearing method on acceptance of deprived and non-deprived young larvae

Next, we quantified how the method of queen rearing influenced the effects of nutritional deprivation on larval selection for emergency queen rearing. Here, we compared two queen-rearing methods – artificial (using plastic queen cups on grafting frames) and natural (on standard brood frames). For each type of queen rearing method, we created groups of deprived and non-deprived larvae, placed them in experimental colonies experiencing emergency queen rearing conditions and then measured how many queens were reared to pupation from each treatment group. When there was no significant difference between replicates, we pooled the data from all replicates within a treatment group for analysis.

**Figure 3:**
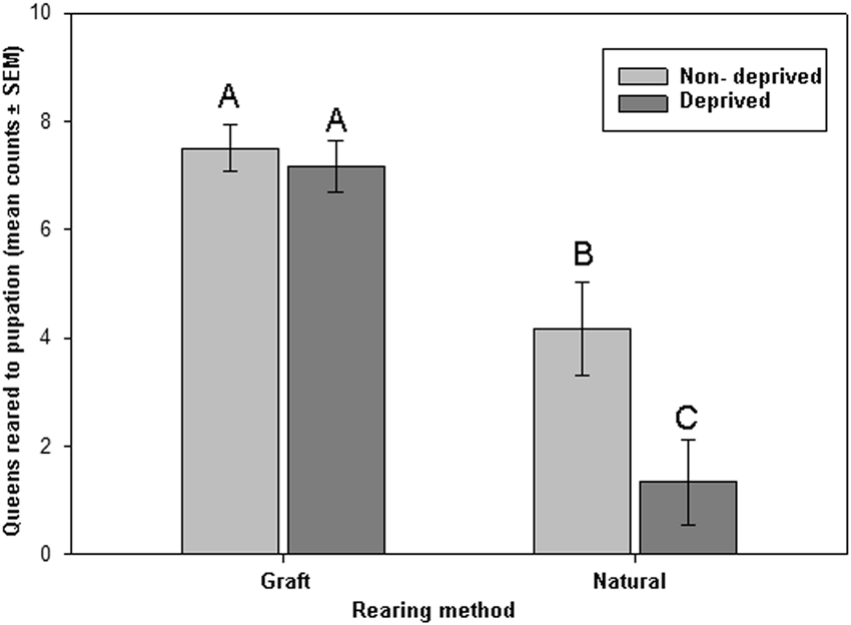
Figure depicts the effects of queen rearing method on the acceptance of deprived (dark grey) and non-deprived (light grey) young larvae for queen rearing. A significantly higher number of larvae were selected for queen rearing (to pupation) from the non-deprived group compared to the deprived group (p < 0.01) when workers selected larvae in the natural selection method. No such significant difference between the groups were observed in the artificial selection method. For all variables with the same letter (A,B or C), the difference between the means is not statistically significant. The data was pooled for all the larvae in each of the deprived and non-deprived groups (N = 2). Bars indicate SEM.

When experimental colonies were allowed to select deprived or non-deprived larvae for queen rearing under the natural emergency queen rearing method, a significantly higher number of queens were reared from the non-deprived treatment group than from the deprived treatment group (χ^2^ = 8.8, df = 1, p < 0.01; Fig. 3). There was no significant difference between the number of queens reared to pupation from deprived and non-deprived larvae, when larvae were grafted into queen cups (grafting method) (χ^2^ = 0.045, df = 1, p = 0.83).

### Experiment 4. Mitigating potential comb odor bias on selection of larvae for queen rearing

In this experiment, we tested whether placing a brood frame in a foster colony for 12 hours could mask the accompanying comb odor sufficiently enough to eliminate any potential bias toward related larvae, that may influence larval selection for queen rearing. To do so, we compared how many queens a colony reared to pupation, from a specific brood area of related totipotent larvae and a brood area of unrelated totipotent larvae, after both had spent 12 hours in unrelated foster colonies.

There was no significant difference observed in the number of larvae selected for queen rearing from the related and unrelated frames (χ^2^ = 0.13, df = 1, p = 0.72) after those frames spent 12 hours in a foster colony. As there were no significant differences observed between the replicates, the data in each treatment group was pooled for analysis.

### Experiment 5. Effects of relatedness and nutritional status on selection of larvae for queen rearing

Finally, we examined how larval nutritional status and the relatedness between worker bees and larvae may interact and affect selection of larvae for queen rearing. We performed this examination once using experimental colonies headed by unrelated, open mated queens, and again with colonies headed by single drone inseminated super-sisters. Within a given experimental set (open mated queen colonies or super-sister queen colonies), we forced all colonies into emergency queen rearing conditions after their queens had laid eggs on experimental brood frames. Then each colony received a single frame of brood that contained four different “types” of larvae: deprived and non-deprived related larvae and deprived and non-deprived larvae from an unrelated colony. We compared the number of queens reared to pupation from these different larval patches. When there was no significant difference between replicates, we pooled the data from all replicates within a treatment group for analysis.

#### Open mated queens

**Figure 4:**
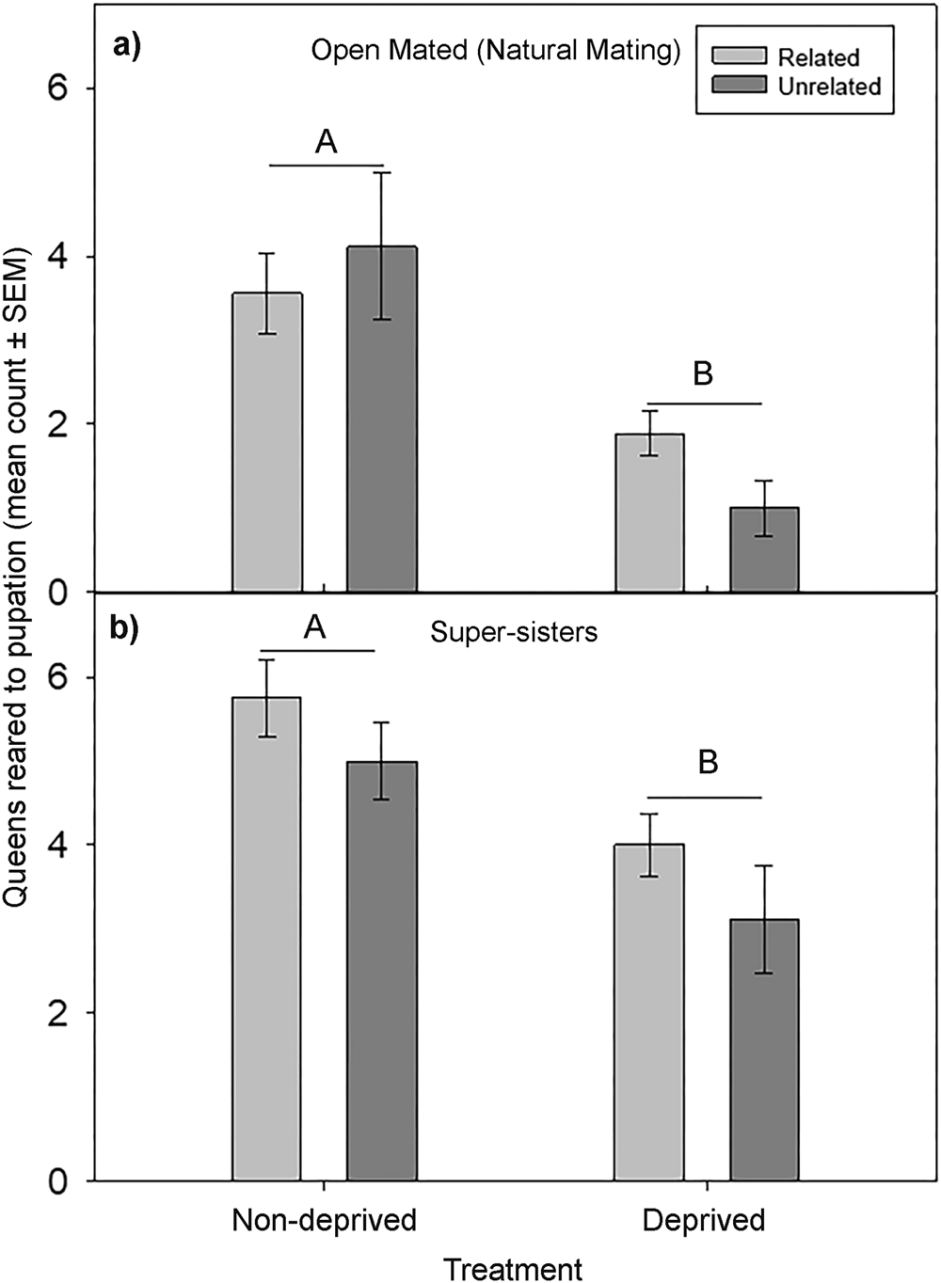
Figure portrays larval selection for queens by workers between related and unrelated colonies having queens which are open mated and queens which are super-sisters (mated by only single drones) based on larval deprivation. No significant difference was observed in the number of queens reared to pupation in both (**a)** open mated (natural mating) and (**b**) super-sister colonies with respect to related (light grey) and unrelated colonies (dark grey). However a significantly higher number of queens were reared to pupation from the non-deprived treatment groups when compared to the deprived group in both (**a**) open mated (p < 0.0001) and (**b**) super-sister colonies (p < 0.05). Bars indicate SEM. N = 2 as the data was pooled for each group. For all variables with the same letter (A or B), the difference between the means is not statistically significant.

Number of queens reared did not significantly differ by relatedness in either the deprived (χ^2^ = 0.86, df = 1, p = 0.35) or the non-deprived larval treatments (χ^2^ = 0.42, df = 1, p = 0.52). However, the number of queens reared differed significantly between larval treatment groups (χ^2^ = 19.46, df = 1, p < 0.0001). For both related and unrelated larvae, more queens were reared to pupation from the non-deprived treatment group (3.8 ± 0.5) than from the deprived treatment group (1.4 ± 0.2; Fig. 4a).

#### Single drone inseminated super-sister queens

Similarly, for larvae from single-drone inseminated super sister queens, it was observed that the number of queens reared to pupation did not differ by relatedness in either the deprived (χ^2^ = 2.46, df = 1, p = 0.12) or the non-deprived larval treatment groups (χ^2^ = 0.36, df = 1, p = 0.55). For both related and non-related larvae, the number of queens reared differed significantly between larval treatment groups (χ^2^ = 5.88, df = 1, p < 0.05), with more queens reared from the non-deprived treatment group (5.4 ± 0.3) than from the deprived treatment group (3.6 ± 0.4; Fig. 4b).

## Discussion

The factors used in the selection of larvae for emergency queen rearing are poorly understood. While it is widely established that larvae are not chosen based on relatedness to workers^47–51^, other factors, for e.g. larval nutrition, health, etc. are poorly elucidated. In this study, we demonstrate that honey bees can perceive the nutritional state of larvae and use such information when selecting larvae for rearing queens in the natural emergency queen replacement process. To our knowledge this is the first study that has thoroughly investigated the role of nutritional state of larvae in selection of larvae for queen rearing.

In a healthy functioning honey bee colony, the queen is the sole reproductive female and is hence crucial for the success of the colony. Robust and high-quality queens are vital for colony growth, survival and reproduction^62–66^. Thus, decisions made during the queen production process are extremely important for colony fitness and are likely driven by selection at the colony level, rather than individual level^65–67^. It is reasonable, therefore, to presume that honey bees possess the ability to assess larval fitness and select the best larvae from which to raise queens. Well-fed larvae are likely of better health and quality, and consequently may develop into healthier, higher-quality queens.

Results from studies investigating worker bee interactions with nest-mates of other castes (specifically virgin queens and young drones) support the notion that workers can evaluate the quality of reproductive individuals and modify their behavior toward them accordingly^67,68^. For example, workers may distribute a greater proportion of their trophallaxis-stimulating vibration signals toward drones with perceived poor flying ability (i.e. lower thorax weight) in order to produce greater numbers of competitive, fit males^68^. In another study, virgin queen survival was positively correlated with worker-queen interactions; and one specific interaction—again, vibration signal—was positively correlated with her fighting ability and number of rivals killed, thereby increasing the chance of a high quality queen becoming the new laying queen of the colony^67^.

Our initial observations suggest that even in a normally functioning colony with adequate resources, some larvae are seemingly stochastically ignored for sufficient time to result in some amount of “hunger.” Even periods of short-term neglect by nurse bees (and a presumed concomitant deprivation of food), in keeping with the natural variation observed in the hive, was sufficient to elicit distinct patterns of nurse bee behavior towards deprived larvae. This variation—not only in nurse bee response times to initiate inspections and feeding bouts, but also in the number of such bouts over the course of an hour-indicates that nurses perceive subtle changes in larval rearing environment and adjust their responses accordingly. The most likely cue for such changes are chemical, though our experimental methodology cannot rule out other cues, as they were not measured in our study. Indeed, research by others have demonstrated that nurse bees can distinguish between well-fed and deprived larvae^35^ and that such differentiation is accomplished by chemical cues^34,36^.

Honey bee larvae have a robust and varied pheromonal profile^69–73^. Brood ester pheromone, for example, has been shown to communicate the presence and number of larvae in a colony, regulating nursing and foraging behaviors over the short- and long-term^72,74–76^. The semiochemical E-β-Ocimene was first described in honey bee queens^77,78^. However, more recent research has demonstrated it to be also emitted by the larvae, for reasons such as to inhibit ovary development in worker bees^79^, accelerate hypopharyngeal gland development in nurse bees^80^ and regulate foraging behavior^81,82^. Perhaps more germane to the study presented here, it has recently been shown that larvae produce more E-β-Ocimene after being deprived of food, lending additional support to the idea that larvae communicate nutritional status chemically^36^. It appears that a deprived larva pheromonally sends a “hunger signal” to positively influence its chances of being fed. Conversely, the same pheromone signal may have a negative effect on the likelihood that worker bees will select the signaling larvae for queen rearing.

In our study (experiments 5), the deprived larvae were not made available to queenless colonies for queen rearing immediately after the deprivation period. Rather, both deprived and non-deprived larvae spent a 12-hour period in foster colonies post-deprivation, and presumably were fed by nurse bees therein, before being placed in the queenless colonies under emergency queen rearing conditions (about 40 hours old at that point). Hence, levels of brood food present in the cells may not have been different enough between the two groups (deprived and non-deprived) to be easily distinguished by workers during the point of time at which they were making decisions to select larvae for rearing queens. It appears that the larvae selected for queen rearing most likely were not chosen based on depleted brood food levels in the larval cells, but more likely based on chemical cues. We speculate that deprived larvae were sending nutritional stress signals in the form of pheromones to workers and workers used those signals while making choices regarding queen rearing. Future work on larval selection for queen rearing should investigate the E-β-Ocimene levels, and other chemical signatures, of larvae that are preferentially selected for queen rearing when nurse bees must choose between well-fed and potentially malnourished larvae.

Beyond transient effects of nurse bee attention, short periods of deprivation can have a profound impact on the trajectory of the life of a larva. As predicted, natural emergency queen rearing and larval grafting methods produced significantly different results. The natural queen rearing method (emergency queen rearing) showed a strong bias toward non-deprived larvae for queen rearing selection, whereas there was no significant difference in the number of queens reared from deprived and non-deprived larvae in the artificial grafting method. Like several previous studies^50,52^, our results support the notion that artificially grafting larvae into queen cells does not appear to be a test for selection, but rather a test for queen rearing maintenance of preselected larvae. The factors that bees use to select larvae for queen rearing and those used to maintain queen larvae may be very different. Likely, in the absence of a queen, the size, orientation and/or shape of queen cells provide a strong signal to nurse bees that larvae contained therein should be reared as queens (copiously provisioned with royal jelly)^17^. In our study, larval nutritional status was a significant factor in selection of larvae for emergency queen rearing under natural conditions, but not for artificially grafted larvae, which were placed inside artificial cups resembling the beginnings of natural queen cells.

The results from the comb odor experiment indicate that workers did not preferentially rear more queens from larvae in their own comb, signifying that a 12-hour period in another colony masks any potential comb odor associated with the parental colony. Most studies that found worker bees selecting related larvae for queen rearing more often than unrelated larvae^38,39,47,52^, did not control for the possibility that the foreign comb odor accompanying unrelated larvae in some way discouraged workers from selecting them. We avoided this methodological pitfall in our experiments on relatedness by placing all experimental brood comb in a foster colony for 12 hours.

Additionally, our findings from the experiment pertaining to relatedness and nutritional state of larvae strongly suggest that nutritional state of larvae is a significant factor in selection of larvae for queen rearing when compared to relatedness. These results further strengthen the existing data demonstrating the absence of nepotism during emergency queen rearing process. Thus, within colonies, the primary factors leading to selection of a larva for a replacement queen appear to be those related to larval fitness, rather than relatedness or origin.

For the past few years, poor queen quality has been a top management concern for commercial beekeeping in the United States^83^. Hence, any new insights about queen production would immensely benefit the beleaguered beekeeping industry. Queen breeders select larvae for queen rearing solely based on age of the larvae^84^ whereas bees may be assessing several other traits in addition to age of larvae when selecting larvae for queen rearing. Our findings illustrate the importance of larval nutrition in selection of larvae for queen rearing.

## Methods

All the experiments were performed during the months of April through September 2008 at Texas A&M University apiary, College Station, TX (30°6′N; 96°32′W). A brief summary of the experiments conducted, their objectives and the sample sizes have been provided in Table 1.

**Table 1:** Table summarizes the rationale behind each of the five experiments that were conducted to establish nutrition-driven larval selection by workers, for queen rearing, in a honey bee colony.

Experiment	Hypothesis/Objective	Sample size	
		*Number of colonies/replicates*	*Number of larvae observed per colony*
Experiment 1	(a) To determine number of larvae (2-day old) that are not fed at all during a four hour observation period and (b) examine variation in nursing times between selected 5-day old larvae	(a) 4 (b) 5	(a) 75 (b) 25
Experiment 2	To compare feeding responses of nurse bees towards deprived and non-deprived larvae of same age	6	6 (deprived larvae) and 6 (non-deprived) larvae per colony
Experiment 3	To investigate how the method of queen rearing (a) natural method versus (b) artificial / grafting method, influences selection of larvae for queen rearing that were either nutritionally deprived or not deprived	(a) 6 (b) 6	(a) All larvae in 340 cm^2^ comb area (b) 12 (deprived larvae) and 12 (non-deprived larvae)
Experiment 4	To examine if 12-hour placement of a frame (comb) with larvae in an unrelated colony would mask the comb odor of the natal colony	6	All larvae in 340 cm^2^ comb area
Experiment 5	To test the effects of relatedness and nutritional status of larvae in selection for queen rearing	(a) 12 (unrelated) (b) 12 (related)	(a) All larvae in 156 cm^2^ comb area (b) All larvae in 156 cm^2^ comb area

The objectives and sample sizes for each experiment have been detailed in the table.

### Experiment 1: Variation in feeding times between same-age larvae

The objective of this experiment was to determine the maximum amount of time any single larva may be neglected by nurse bees and the amount of time nurse bees spend feeding each larva.

#### Young larvae

Four colonies were established in separate four-frame observation hives, each containing a queen, approximately 4,000 adult honey bees, and equal amounts of brood, honey, and pollen. In each of the four observation hives, we video recorded a randomly selected brood area consisting of 75 neighboring, two-day old larvae for four consecutive hours, using a digital camera (Sony, New York, NY, USA). The four-hour observation period was chosen based on previous studies^85^. We analyzed these videos to determine the number of larvae that were not visited by nurse bees during the 4-hour observation period.

#### Older larvae

Five colonies were established in separate two-frame observation hives, each containing a queen, approximately 2,000 adult honey bees, and equal amounts of brood, honey, and pollen. In each of the observation hives, we video recorded five randomly chosen five-day old larvae for one hour with a digital camera. This was repeated five times for a total of 25 larvae observed per colony. Next, we analyzed the videos for the duration of nurse bee feeding bouts for each larva. A feeding bout was defined as a nurse bee inserting her head and thorax into the larval cell^86^ for three or more seconds. We then summed the amount of time nurse bees spent feeding each larva to estimate total time fed during the recorded time. The variance of total feeding times among the larvae was tested against a uniform distribution with Student’s T-test^87^ using SPSS Statistics (2008) 16.0.

### Experiment 2. Feeding response to nutritionally deprived and non-deprived larvae

Six colonies were established in separate four-frame observation hives as described above for 1a. In each colony, we confined the queen in a 340 cm^2^ area (henceforth referred to as target area) on one side of a frame using a push-in cage constructed of 3 mm hardware cloth on the sides and plastic queen excluder material on top (Supplementary Fig. 1). The queen was allowed to lay eggs in this area for approximately 24 hours. After 24 hours, we removed the queen from the target area and caged her on another frame in the same hive to prevent further egg laying in the target area.

When larvae in the target area were 24 hours old, we enclosed two equal-sized, side-by-side sections of the target area, each containing approximately 500 larvae, with two different 169 cm^2^ hardware cloth cages pushed into the wax surrounding the larvae. The cages were made of different mesh sizes: we constructed one from 13 mm hardware cloth, which did not impede adult bee access to larvae (allowing that side to serve as a non-deprived control) and made the other with 3 mm hardware cloth, which restricted adult bee access to larvae (depriving larvae of food) (Supplementary Fig. 2). The cages stayed on for four hours. After four hours, we removed the hardware cloth cages from the target area.

We randomly selected six larvae from each treatment area (deprived and non-deprived) and video recorded them with a digital camera for 30 minutes. These videos were analyzed for nurse bee feeding bouts and cell inspections (as defined in Experiment 1). We measured the following parameters for each larva: time to first feeding, time to first inspection, total number of feeding bouts, total number of inspections, duration of each feeding bout and total time fed.

Time to first feeding bout and time to first inspection were log-transformed and analyzed by ANOVA^87^. Total number of feeding bouts and total number of inspections were analyzed by Chi-squared test^87^. Mean feeding bout duration was analyzed by Mann-Whitney U test and total feeding time was analyzed by ANOVA^87^. We performed all statistical analyses using SPSS Statistics (2008) 16.0.

### Experiment 3. Effect of queen rearing method on acceptance of deprived and non-deprived young larvae

#### Natural emergency queen rearing method

Six healthy colonies each with approximately 40,000 bees, each housed in their own standard, two-story Langstroth hives, were used for this experiment. We confined the queen of each colony in a 340 cm^2^ area (target area) on one side of an empty, drawn-out frame, using a push-in cage as described above (Experiment 2) and allowed her to lay eggs for approximately 12 hours. After 12 hours, we removed the queen from the target area and caged her on another frame to prevent further egg laying in the target area.

The eggs were allowed to hatch naturally in the colony and when the larvae in the target area were one day old, we applied two push-in cages to the target area to create a section of larvae that had been deprived for 4 hours as described above (Experiment 2). Twelve hours after removal of the push-in cages, we moved the frames (now containing an approximately equal number of deprived and non-deprived larvae) from each of the six experimental colonies into six unrelated colonies experiencing natural, emergency queen rearing conditions (made queenless and broodless 24 hours earlier). We then counted the number of queens reared to pupation from the deprived and non-deprived areas of larvae in each colony and compared them by Chi-squared test^87^ using SPSS Statistics (2008) 16.0. As there was no significant difference between replicates, we pooled the data from all replicates within a treatment group for analysis.

#### Artificial larval grafting method

For the artificial grafting portion of the experiment, we transferred the deprived and non-deprived larvae obtained from six experimental colonies as described above (Experiment 3 a) to commercially available, vertically-oriented, plastic queen cups (M00664 Wide base Cell Cups, Dadant & Sons, Hamilton, Illinois) that were fitted to slotted bars positioned horizontally in a foundation-less, comb-less frame. For each of six experimental colonies, we grafted twelve deprived and twelve non-deprived larvae into the plastic queen cups of one grafting frame and then placed the grafting frames in six different unrelated, queenless and broodless colonies as described above (Experiment 3 a). Cups containing deprived and non-deprived larvae alternated along each grafting bar of a grafting frame. We counted the number of queens that were reared to pupation and analyzed these data by Chi-squared test^87^ using SPSS Statistics (2008) 16.0. As there was no significant difference between replicates, we pooled the data from all replicates within a treatment group for analysis.

### Experiment 4. Mitigation of comb odor bias on selection of larvae for queen rearing

Six five-frame nucleus colonies, each containing a queen, approximately 5,000 adult bees, one pollen frame, one honey frame and three empty drawn frames (including one split frame) were established. A split frame is a whole frame, cut vertically down the middle, then rejoined with screws at the top and bottom bars for easy disassembly (Supplementary Fig. 3). In each of the six colonies, we confined the queen in a 340 cm^2^ area (target area) on one side of a split frame using a push-in cage, and allowed her to lay eggs for approximately 12 hours. After 12 hours, we removed the queen from the target area and caged her on another frame to prevent additional egg laying in the target area.

We grouped the six colonies into three pairs of two. When the eggs hatched and larvae were 24 hours old, we removed the split frames from each pair of colonies and placed them in a two-story foster colony (one foster colony for each pair of split frames). This enabled the larvae in the split frames to acquire the odor of the foster colonies. To stimulate queen rearing conditions, we also removed the queens from all experimental nucleus colonies at this time. After 12 hours in the foster colonies, half of the split frame from a given nucleus colony was joined with half of the split frame from its paired colony and vice versa. After swapping respective halves like this, we placed the new combinations of split frames back in the paired, now queenless, colonies (Supplementary Fig. 4). After introduction, we inspected the split frames every 24 hours for development of queen cells and recorded the number of queens reared to pupation. We analyzed the data by a Chi-squared test^87^, using SPSS Statistics (2008) 16.0. As there was no significant difference between replicates, we pooled the data from all replicates within a treatment group for analysis.

### Experiment 5. Effects of relatedness and nutritional status on selection of larvae for queen rearing

#### Open mated queens

To test the effects of relatedness and nutritional status on larval selection for queen rearing in colonies headed by open mated queens, we established twelve five-frame nucleus colonies. We confined the queens in each of these colonies on split frames for 12 hours as described above (Experiment 4). When the larvae were 24 hours old, we covered half the brood in the target area on one half of a given split frame with a push-in 3 mm hardware cloth cage (approx. 78 cm^2^ area), so that adult bees did not have access to the larvae (deprived larvae). We covered the other half of the target area on that half of the split-frame with a push-in 13 mm hardware cloth cage (approx. 78 cm^2^ area) that gave nurse bees free access to larvae (non-deprived larvae) (Supplementary Fig. 5). We then placed the split frames back in their colonies. Four hours later, we removed the push-in cages and placed the split frames in designated foster colonies to mitigate potential comb odor bias as described above (Experiment 4). In order to stimulate queen rearing behavior, experimental nucleus colonies were dequeened at this time. After 12 hours, we removed the frames from foster colonies and swapped split frame halves between a pair of nucleus colonies as described above (Experiment 4). We then recorded the number of queens reared to pupation on each of the four larval patches (two deprived and two non-deprived) on the split frames in all experimental colonies (Supplementary Fig. 6). Chi-squared test^87^ was performed to analyze the data, using SPSS Statistics (2008) 16.0. As there was no significant difference between replicates, we pooled the data from all replicates within a treatment group for analysis.

#### Single drone inseminated super-sisters

To test the effects of relatedness and nutritional status on larval selection for queen rearing in colonies headed by single drone inseminated super-sister queens, we followed similar methods as described above (Experiment 5 a). However, the queens used in this experiment were super-sisters (same mother and same father; genetic relatedness of 0.75) that were each inseminated with semen from a different single drone; whereas the queens used in the previous experiment were open mated. The super-sister queens for this experiment were obtained from Glenn Apiaries, CA. We established these experimental super-sister queen colonies two months before the initiation of the experiment to make sure that the worker bee population at the initiation of the experiment were progeny of the sister-queens. Chi-squared test^87^ was performed to analyze the data, using SPSS Statistics (2008) 16.0. As there was no significant difference between replicates, we pooled the data from all replicates within a treatment group for analysis.

### Data availability

All data generated or analyzed during this study are included in this published article (and its Supplementary Information files).

## Electronic supplementary material

Supplementary Information
