# Supplementary material for: Honey bees consider larval nutritional status rather than genetic relatedness when selecting larvae for emergency queen rearing
Source: Sci Rep. 2018 May 16;8:7679. doi: 10.1038/s41598-018-25976-7 (PMC5955998; doi:10.1038/s41598-018-25976-7)
Supplement: Supplementary file 1 — Supplementary Information [file 41598_2018_25976_MOESM1_ESM.pdf]

Supplementary Information

Honey bees consider larval nutritional status rather than genetic relatedness when selecting larvae for emergency queen rearing

Ramesh R Sagili, Bradley N Metz, Hannah M Lucas, Priyadarshini Chakrabarti, Carolyn R Breece

Correspondence to: ramesh.sagili@oregonstate.edu

Supplementary Fig. 1

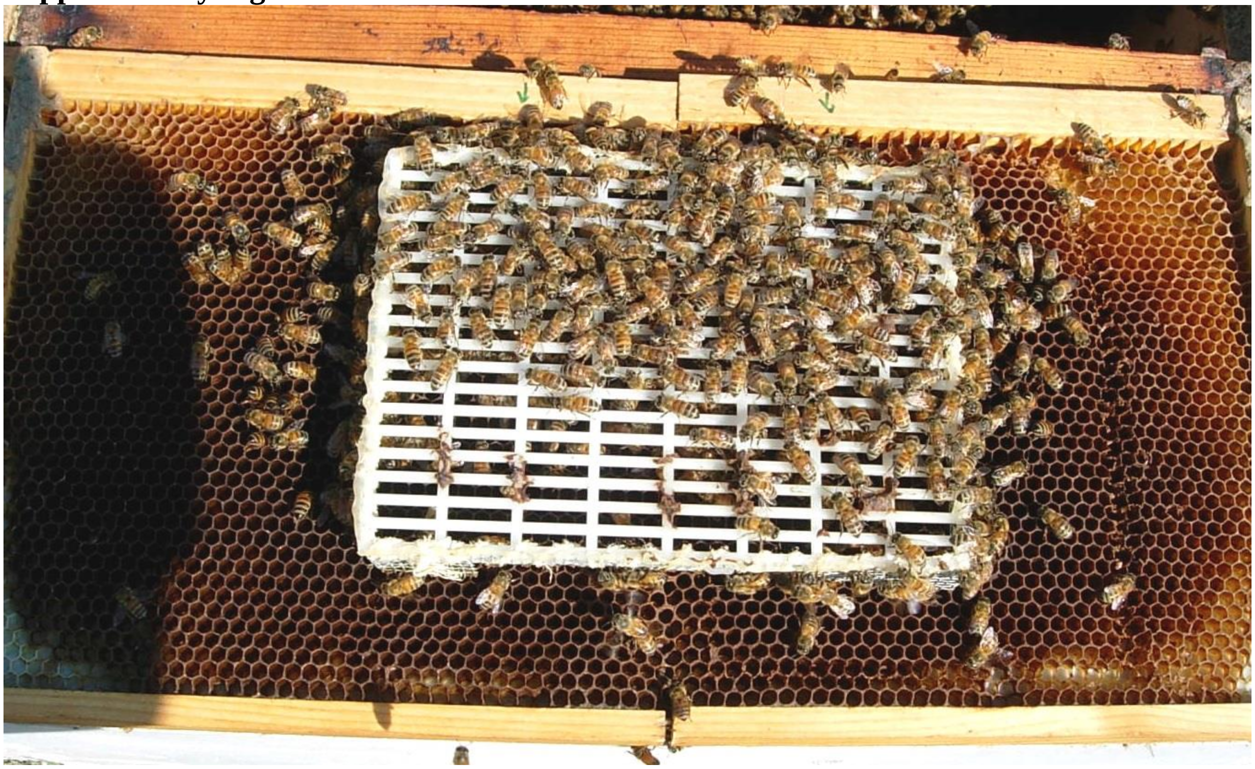

A representative frame showing the queen excluder used in all the experiments. The queen was confined in the target region of the frame using push-in cage constructed of 3 mm hardware cloth on the sides and plastic queen excluder material on top.

Supplementary Fig. 2

Deprived (3 mm mesh width)

Non-deprived (13 mm mesh width)

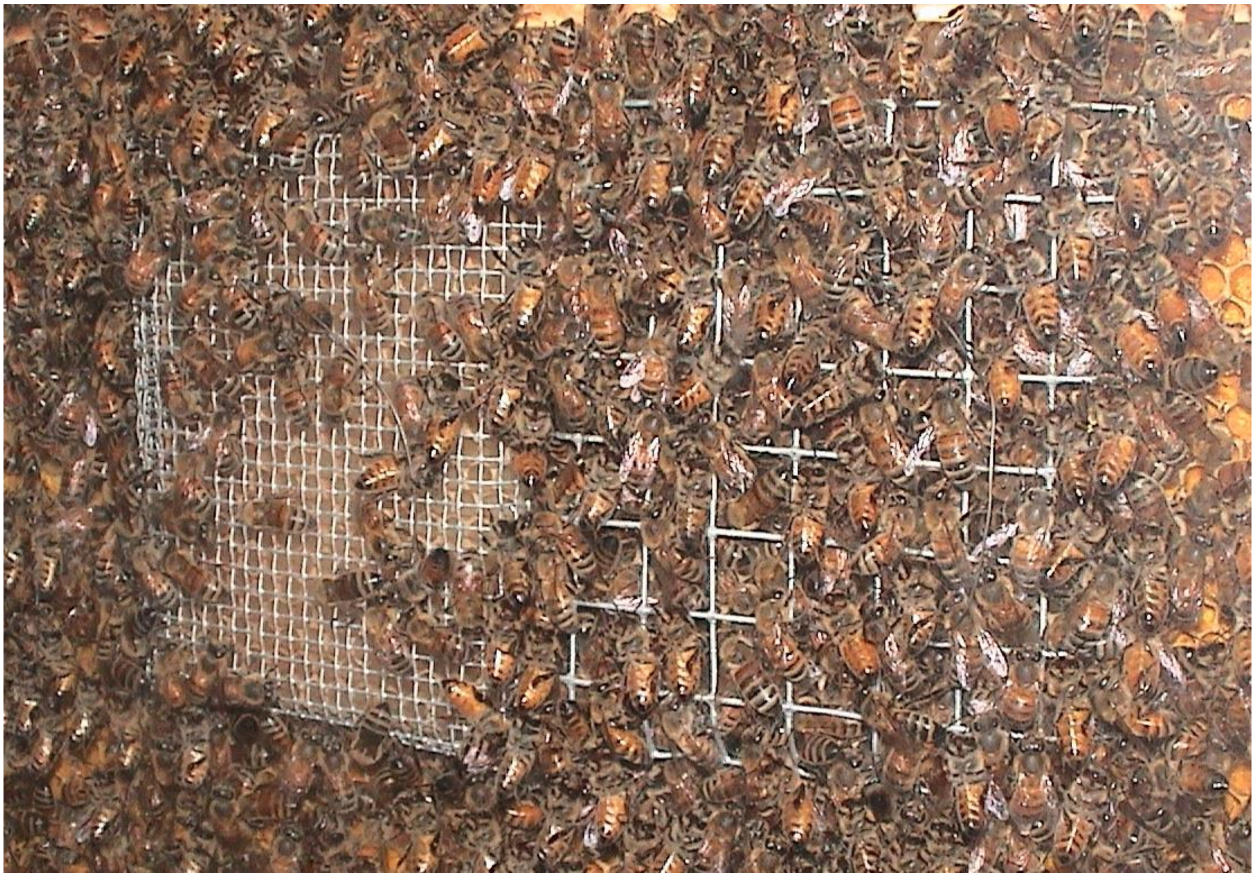

A representative frame showing the two mesh sizes of hardware cloth used for the two larval groups – deprived and non-deprived.

Supplementary Fig. 3

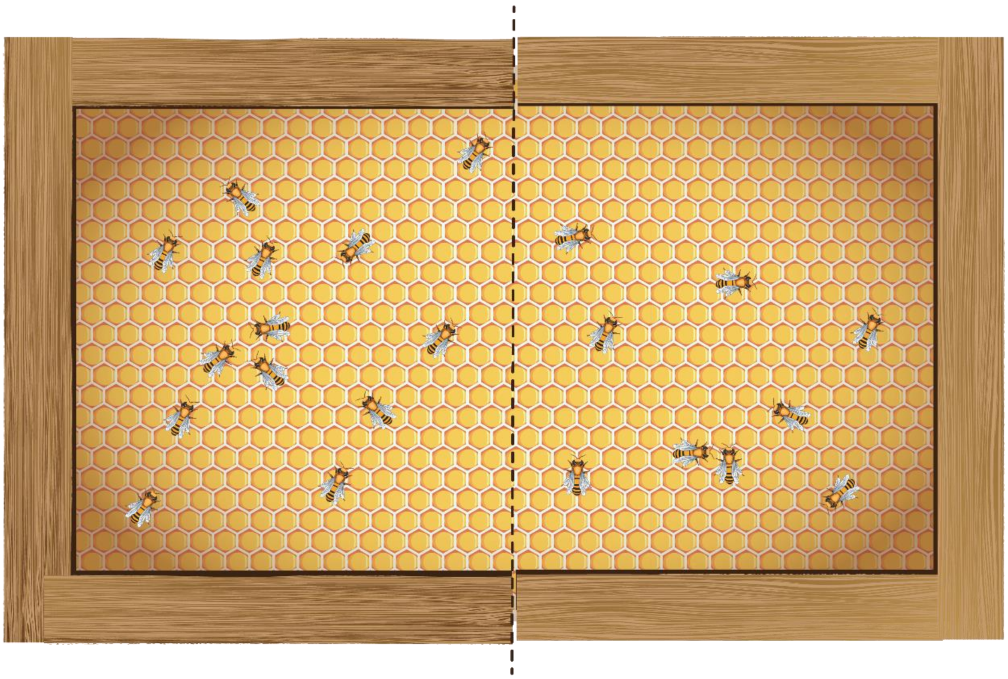

A representative split frame. The dotted line indicates the region where the two frame halves can be separated and joined.

Supplementary Fig. 4

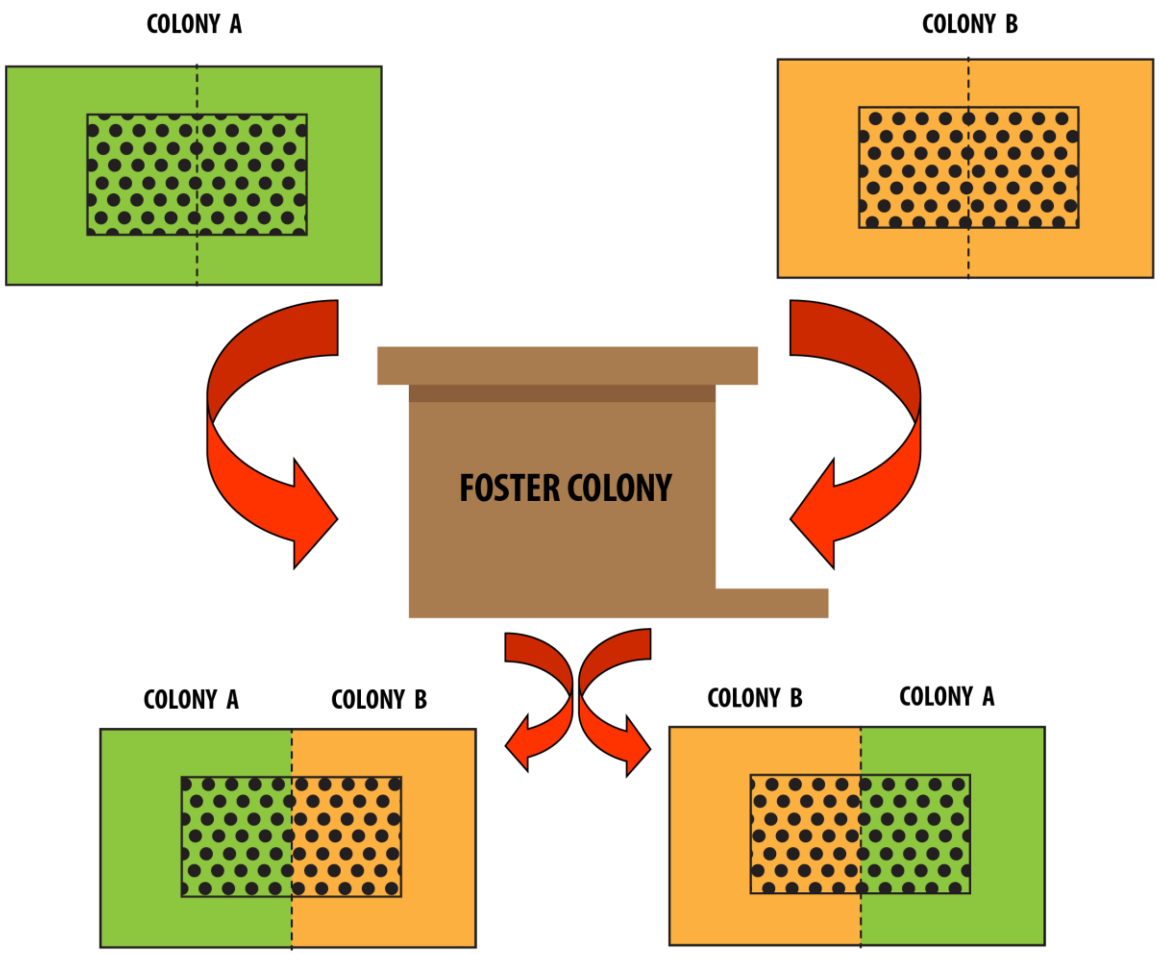

The experimental design to test effects of comb odor. A queen laid eggs in target area (polka dotted) of split frame (separable at dotted line); two experimental colonies were paired together (A and B); split frames were placed in foster colonies for 12 hours; then halves of the split frames were swapped between paired colonies and placed back in A and B.

Supplementary Fig. 5

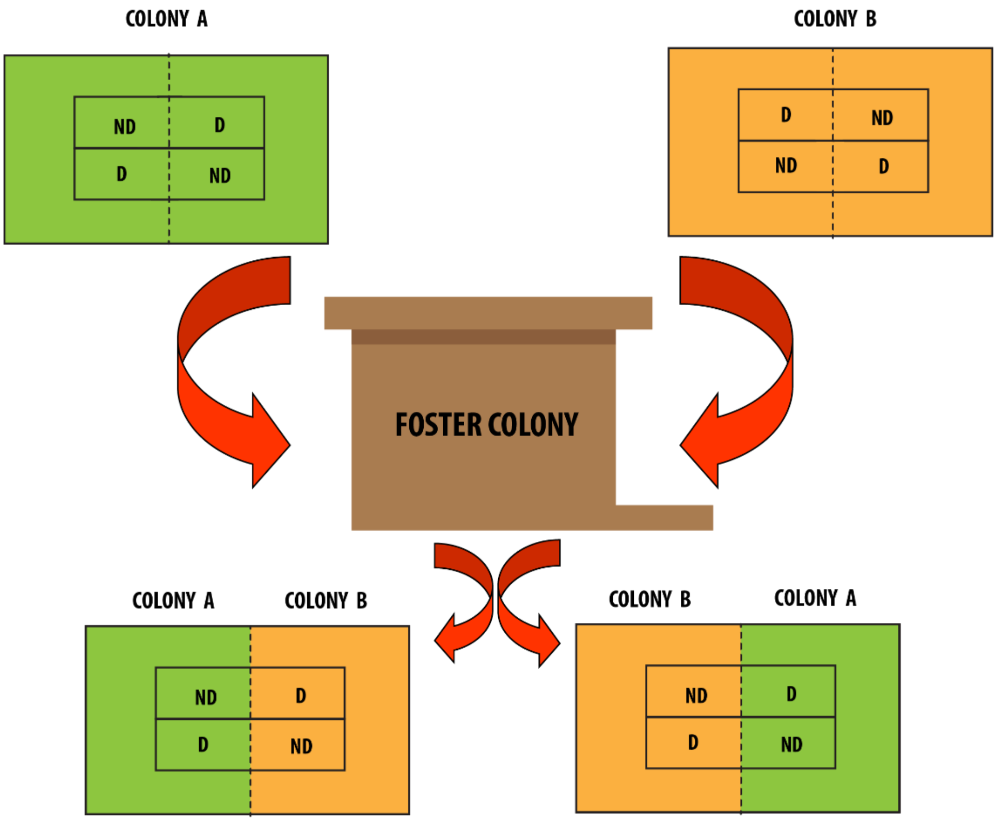

The experimental design for establishment of deprived and non-deprived larvae on split frames. A queen laid eggs in target area (polka dotted) of split frame (separable at dotted line); two experimental colonies were paired together (A and B); on each separate half of a split frame, 50% of larvae were covered with a cage depriving them of nurse bee attention and food (D, deprived) and 50% of the larvae were covered with a cage that allowed nurse bees to feed them (ND, non-deprived); after four hours, cages were removed and frames were placed in foster colonies for 12 hours; then halves of the split frames were swapped between paired colonies and placed back in A and B.

Supplementary Fig. 6

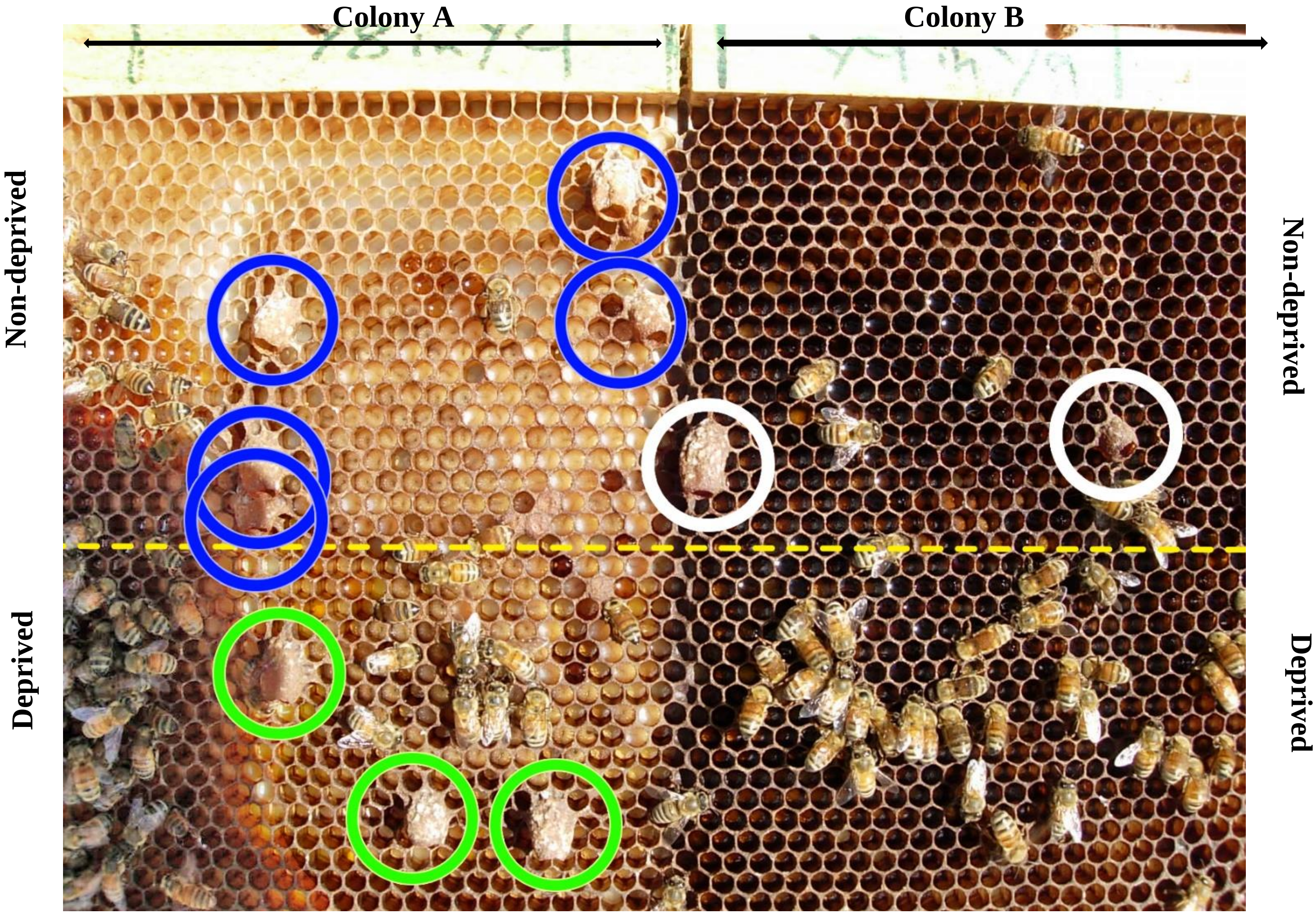

A representative frame showing the number of queen cells formed. This is indicative of the number of larvae selected by workers for queen rearing up to pupation between the deprived and the non-deprived halves of two spilt frames. These two halves joined together to form a whole frame are from a pair of nucleus colonies (colonies A and B) as has been described in the text. Blue circles indicate queen cells reared in the non-deprived region of colony A whereas green circles indicate queen cells reared from the deprived region. White circles indicate queen cells reared from the non-deprived region of colony B whereas the deprived region shows no queen cell formation. Yellow dotted line indicates the region of separation between the deprived and non-deprived areas for both the halves.
